# High Sensitivity and Selectivity of PEDOT/Carbon Sphere Composites for Pb^2+^ Detection

**DOI:** 10.3390/molecules30040798

**Published:** 2025-02-09

**Authors:** Lirong Ma, Zhuangzhuang Wang, Xiong Liu, Feng Xu, Tursun Abdiryim

**Affiliations:** State Key Laboratory of Chemistry and Utilization of Carbon Based Energy Resources, College of Chemistry, Xinjiang University, Urumqi 830017, Chinaliuxiong@xju.edu.cn (X.L.)

**Keywords:** Poly(3,4-ethylenedioxythiophene), carbon sphere, electrochemical sensors, Pb^2+^ detection

## Abstract

Heavy metal ions impair human health and irreversibly damage the ecosystem. As a result, it is critical to create an efficient approach for identifying heavy metal ions. The electrochemical sensor method is a type of detection method that is highly sensitive, low in cost, and allows for real-time monitoring. In this study, solid carbon spheres were made using resorcinol and formaldehyde as raw materials, followed by the formation of PEDOT/carbon sphere composites via in situ oxidative polymerization, and Pb^2+^ was detected utilizing them as electrode modification materials. The structure of the PEDOT/carbon spherical composites was analyzed using scanning electron microscopy (SEM), X-ray diffraction (XRD), and Fourier transform infrared spectroscopy (FTIR). To investigate the electrochemical properties of these composites, electrochemical impedance spectroscopy (EIS), cyclic voltammetry (CV), and differential pulse voltammetry (DPV) were employed. In addition, the detection mechanism of the material for Pb^2^⁺ was studied using CV. The PEDOT/carbon sphere sensor showcased an extensive linear detection range of 7.5 × 10^−2^ to 1.0 μM for Pb^2+^ ions, achieving a low limit of detection (LOD) of 3.5 × 10^−2^ nM and displaying exceptional selectivity. These results can be attributed to its large surface area, superior electrical conductivity, and outstanding electron transport properties. This study offers an effective material for detecting low concentrations of Pb^2+^, with potential applications in future Pb^2+^ detection.

## 1. Introduction

With the rapid advancement of industrialization, pollution of soil and groundwater by heavy metals has emerged as one of the most significant environmental issues globally [1]. Heavy metal ions are not only non-degradable but can also enter and accumulate in the human body through skin contact, diet, and respiration, thus causing a series of health problems, including organ damage, chronic poisoning, and nervous system disorders, and leading to the continuous reduction in drinking water resources worldwide [2]. In particular, lead ions (Pb^2+^) not only directly interfere with the function of the immune system but also have toxic side effects on the nervous system and the kidneys [3,4,5] and affect the growth and development of water bodies, soils, and plants [6,7]. Consequently, it is essential to create an analytical method that is rapid, sensitive, and straightforward for the detection of heavy metal ions. This is crucial for safeguarding human health, ensuring ecological balance, and advancing sustainable development.

According to the available literature, several methods have been employed to identify Pb^2+^, including atomic absorption spectrometry [8,9], colorimetry [3,10], and inductively coupled plasma mass analysis [11,12]. Even though these techniques possess great precision and sensitivity, they are more complicated and cumbersome in the sample pretreatment process and instrument operation. In recent years, 999 electrochemical sensors have been frequently employed for detecting heavy metal ions, owing to their numerous advantages, such as simple operation, high sensitivity, low detection limits, and fast analysis times [13,14,15]. The analytical capability of electrochemical sensors primarily relies on the adsorption properties of electrode modification materials, and excellent adsorbability can significantly improve the detection efficiency of analytes [16,17]. Therefore, it is important to explore and develop efficient materials for modifying electrodes in order to improve the detection capabilities of heavy metal ions. At present, researchers have created numerous materials to enhance the functionality of electrochemical sensors, including metals and metal oxides [18], graphene [19], perovskites [20], and polymer-based substrate materials [21,22].

Due to their remarkable electrical conductivity, high chemical stability, and outstanding biocompatibility, conducting polymers have attracted considerable attention [23,24]. Among them, Poly(3,4-ethylenedioxythiophene) (PEDOT) is particularly noteworthy, owing of its outstanding electrical conductivity, optical transparency, and stability, along with its controllable electrochemical properties [25]. These characteristics make PEDOT an ideal candidate for various applications, such as flexible electronics [26], photovoltaic cells [27], and advanced sensors [28,29,30]. Despite these advantages, PEDOT often faces challenges such as agglomeration, which can severely diminish its electroactive sites and compromise its electrochemical performance [31]. Addressing this issue requires the development of strategies or materials that can effectively leverage and enhance PEDOT’s inherent properties to overcome its limitations. A carbon sphere has the characteristics of a high specific surface area, corrosion resistance, high temperature stability, high conductivity, excellent catalytic activity, and good chemical stability [32,33]. It can be used as a carrier to uniformly load PEDOT on the surface of carbon spheres, reduce the agglomeration phenomenon of PEDOT, and thus improve its electrochemical activity and stability. At the same time, its structure also contains hydroxyl functional groups [34], which can form non-covalent bonds with sulfur atoms in the main chain of PEDOT, resulting in adsorption and enrichment of Pb^2+^ and thus achieving a high response to Pb^2+^.

In this research, we effectively formulated and synthesized a PEDOT/carbon sphere composite through in situ polymerization. Then, it was utilized as an electrochemical sensor (PEDOT/carbon sphere/GCE) for detecting Pb^2+^ ions. During the preparation, PEDOT was combined with carbon spheres, which act as a supporting framework, allowing the PEDOT to be uniformly dispersed throughout the composite. The interaction of π-π stacking between PEDOT and carbon spheres strengthens their connection, which in turn minimizes the agglomeration of PEDOT and improves the stability of the composite material. Additionally, the high surface area of the carbon spheres offers more active sites, further improving the electrochemical efficiency of the PEDOT/carbon sphere composite. The presence of hydroxyl groups (-OH) on the surface of the carbon spheres facilitates the formation of non-covalent interactions with the sulfur atoms in the PEDOT chain, thereby improving the composite’s sensitivity and selectivity towards Pb^2+^ ions. To thoroughly analyze the composite’s properties and examine its morphology and structure, we employed scanning electron microscopy (SEM), X-ray diffraction (XRD), and Fourier transform infrared spectroscopy (FTIR). We also investigated how varying the ratios of PEDOT to carbon spheres, pH levels, and deposition times affected the composite’s properties. A comprehensive analysis of the PEDOT/carbon sphere/GCE sensor’s electrochemical behavior, including its linear range, detection limits, and selectivity, was conducted. These findings confirm the effectiveness of PEDOT/carbon sphere composites in Pb^2+^ detection and provide a solid foundation for future applications and research.

## 2. Result and Discussion

### 2.1. Structural Characterization

**Figure 1:**
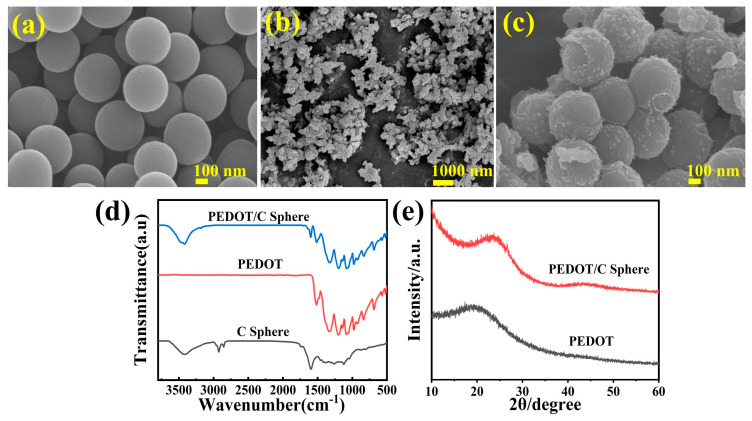
SEM images of (**a**) C sphere, (**b**) PEDOT, and (**c**) PEDOT/C sphere (5:1). (**d**) FTIR spectra of C sphere, PEDOT, and PEDOT/C sphere. (**e**) XRD patterns of PEDOT and PEDOT/C sphere.

Figure 1a–c show SEM images of solid carbon spheres, PEDOT, and PEDOT/carbon sphere composite material. Figure 1a reveals that the solid carbon spheres exhibit a uniform spherical shape. Figure 1b demonstrates that PEDOT displays an irregular aggregation distribution, which is attributed to the aggregation of PEDOT during formation, leading to a reduced surface area and, consequently, decreased electrochemical activity and active sites. Figure 1c illustrates the morphology of the PEDOT/carbon sphere composite, showing that PEDOT was successfully distributed on the surface of the solid carbon spheres due to the interaction between PEDOT and the carbon spheres. This composite approach significantly reduces PEDOT aggregation, providing more active sites and enhancing PEDOT’s chemical stability and electrochemical activity. The hydrogen bond between the -OH groups on the solid carbon spheres and oxygen atoms in PEDOT and increases the stability and activity of the composite material [35].

Figure 1d illustrates the FTIR spectra of the PEDOT/carbon ball composite, PEDOT, and carbon sphere. PEDOT exhibits distinctive peaks at 1515 cm⁻^1^ and 1329 cm⁻^1^, which correspond to the main chain vibrations of C-C and the stretching vibrations of C-C, respectively. These peaks indicate the characteristic vibrational modes of the polymer [36]. Both the carbon sphere and the PEDOT/carbon sphere composite show an absorption peak at 3450 cm^−1^, which is associated with the stretching vibrations of -OH groups. This peak highlights the presence of hydroxyl functionalities in the materials. The peak at 1600 cm^−1^ in the PEDOT/carbon sphere composite could be due to bending vibrations arising from the interaction between the ether bonds in PEDOT and the -OH groups in the carbon sphere. The absorption spectra of the PEDOT/carbon sphere composite and PEDOT reveal peaks at 1515 cm^−1^, 1309 cm^−1^, 1189 cm^−1^, 1137 cm^−1^, 1088 cm^−1^, 1048 cm^−1^, 974 cm^−1^, 920 cm^−1^, 830 cm^−1^, and 660 cm^−1^. Notably, the absorption peaks at 1515 cm^−1^ and 1309 cm^−1^ correspond to the stretching vibrations associated with C=C and C-C bonds found in the thiophene ring structure [37]. The absorption peaks observed at 1189 cm^−1^, 1137 cm^−1^, 1088 cm^−1^, and 1048 cm^−1^ are ascribed to the bending vibrations of C-O-C within the subethoxy group [38]. Additionally, the peaks observed at 974 cm^−1^, 920 cm^−1^, 830 cm^−1^, and 660 cm^−1^ are related to the vibrational modes of the C-S-C bonds within the thiophene ring structure [39].

Figure 1e shows the XRD images of the PEDOT/carbon sphere composites and PEDOT. It can be seen from the figure that both show a wide diffraction peak in the range of about 2θ = 20~25°, which is due to the amorphous structure that is generated by the internal π−π* stacking of PEDOT and PEDOT/carbon sphere composites. The diffraction peak of the PEDOT/carbon sphere is shifted, because the diffraction peak of the carbon sphere appears at the position of 2θ = 20° after PEDOT is combined with the carbon sphere.

### 2.2. PEDOT/Carbon Sphere Electrochemical Behavior Analysis

**Figure 2:**
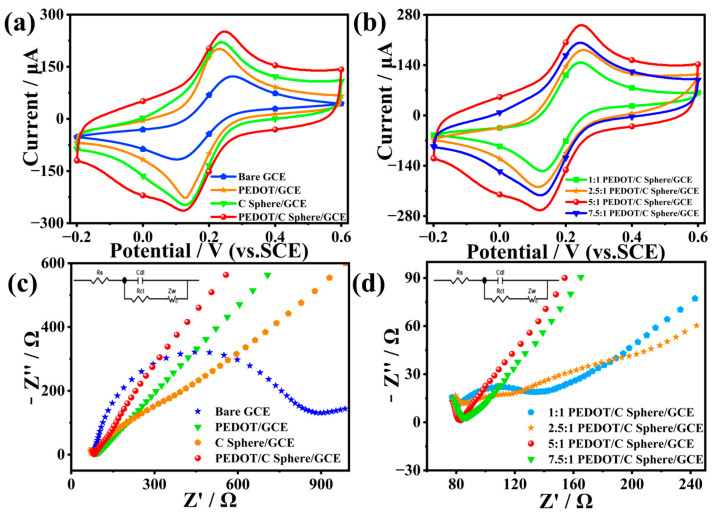
CV of different materials (**a**). CV of PEDOT/C spheres with different proportions. (**b**) EIS of different materials. (**c**) EIS of PEDOT/C spheres with different proportions (**d**).

To investigate the electrochemical interactions between different surface-modified electrodes and electrolyte solutions, cyclic voltammetry (CV) measurements were performed in a 0.1 mol·L^−1^ KCl solution supplemented with 5 mmol·L^−1^ K_3_[Fe(CN)_6_]. Figure 2a demonstrates that the cyclic voltammetry (CV) curves for the different materially modified electrodes indicate that both the PEDOT/GCE and carbon sphere/GCE exhibit greater oxidation-reduction peak currents compared to the unmodified GCE. This enhancement can be attributed to the improved conductivity and electrochemical activity of the PEDOT and carbon spheres. The response current is maximized when PEDOT is combined with carbon spheres, owing to their greater electroactive surface area and enhanced electron transfer efficiency. Figure 2b presents the CV curves of PEDOT/carbon sphere composite materials at different ratios (1:1, 2.5:1, 5:1, 7.5:1). It can be observed that all four ratios show distinct oxidation-reduction peaks within the range of 0.16~0.26 V, with the highest peak current being recorded for the 5:1 PEDOT/carbon sphere composite material. This suggests that the electron transfer capabilities vary with different ratios of PEDOT when combined with carbon spheres in a composite state. Furthermore, the impedance spectroscopy (EIS) curves for different electrode modification materials are depicted in Figure 2c. The impedance spectrum features a semicircular section that represents electron transfer resistance at high frequencies, while the linear section reflects diffusion processes occurring at lower frequencies. The Nyquist curves (Figure 2c,d) were recorded at an open circuit voltage and in the equivalent simulated circuit model (Rct: charge transfer resistance; Zw: Warburg impedance; Rs: solution resistance; Cdl: double-layer capacitance).

The results demonstrate that both pure PEDOT or pure carbon-sphere-modified electrodes exhibit reduced charge transfer resistance compared to bare electrodes, indicating accelerated electron transfer rates upon modification with either material alone. PEDOT/carbon sphere composite materials display even lower charge transfer resistance values than pure PEDOT or carbon spheres individually, suggesting higher electrochemical activity and conductivity of the composites. This finding aligns with the results that were obtained from CV analysis. Figure 2d shows the EIS curves of the PEDOT/carbon sphere composites with different proportions (1:1, 2.5:1, 5:1, 7.5:1). Figure 2d indicates that the charge transfer resistance values for the 1:1 and 2.5:1 PEDOT/carbon sphere composites are greater, whereas the values for the 7.5:1 PEDOT/carbon sphere composite are lower. Compared with other ratios, 5:1 PEDOT/carbon sphere composites exhibit higher electrochemical activity and conductivity with a lower charge transfer resistance. The 5:1 PEDOT/carbon sphere composite is the best electrode modification material. Therefore, the 5:1 PEDOT/carbon ball composite material was selected as having the best proportions of composites as the electrode modification material for the next test.

### 2.3. Optimization of Test Condition for Pb^2+^ Analysis

**Figure 3:**
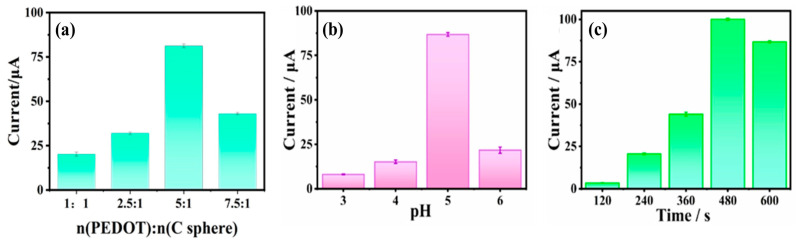
The effects of different ratios of PEDOT to carbon sphere on the detection of Pb^2+^ peak current. (**a**) The effect of pH on the detection of the Pb^2+^ peak current. (**b**) The effect of deposition time on the detection of the Pb^2+^ peak current by the composite-modified electrode (**c**).

For PEDOT/carbon sphere composites, the molar ratio of PEDOT to carbon sphere has a significant impact on the recognition ability of Pb^2+^, as illustrated in Figure 3a. The figure indicates that the sensor’s current response is greatest when the molar ratio is 5:1. When the ratio is less than 5:1, the interaction between the composite material and Pb^2+^ is insufficient, resulting in a decrease in the number of Pb^2+^ recognition sites on the membrane. When the ratio is greater than 5:1, the amount of PEDOT is too large, and agglomeration occurs, resulting in a decrease in the sensitivity of the sensor to detect Pb^2+^. Therefore, the optimal molar ratio of PEDOT to carbon spheres is 5:1.

pH is one of the important factors affecting the sensor’s determination of Pb^2+^. Consequently, PEDOT/carbon sphere/GCE was utilized to investigate how the pH affects the current response of Pb^2+^ in an ABS solution, specifically within a pH range of 3.0 to 6.0. The effect of pH on the peak current was examined using differential pulse voltammetry (DPV) in an ABS solution with 1 μM Pb^2+^. As shown in Figure 3b, the current response changes as the pH value increases. When the pH is 5.0, the electrochemical response signal reaches the highest level, because the binding strength of the Pb^2+^ and PEDOT/carbon sphere composite varies with the pH value, and the interaction between Pb^2+^ and composite material is different at different pH values. As a result, an ABS solution at a pH of 5.0 was selected as the medium for the next electrochemical testing.

The deposition time is a crucial factor affecting the sensor’s determination of Pb^2+^. Therefore, we conducted deposition of PEDOT/carbon spheres/GCE in a solution of 1.0 μM Pb^2+^ in ABS (pH 5.0) for times ranging from 120 to 600 s, recording every 120 s, to assess the impact of the deposition time on the Pb^2+^ current response. As illustrated in Figure 3c, the current response fluctuated with the deposition duration. Remarkably, when the deposition duration reached 480 s, the electrochemical response signal peaked. Beyond 480 s, the current response of Pb^2+^ slightly decreased, indicating that Pb^2^⁺ adsorption onto the electrode surface had attained saturation. Extended deposition times did not substantially influence the amount of Pb^2^⁺ that was adsorbed on the surface of the modified electrode. Thus, 480 s was determined to be the optimal duration for deposition.

### 2.4. Electrochemical Mechanism

**Figure 4:**
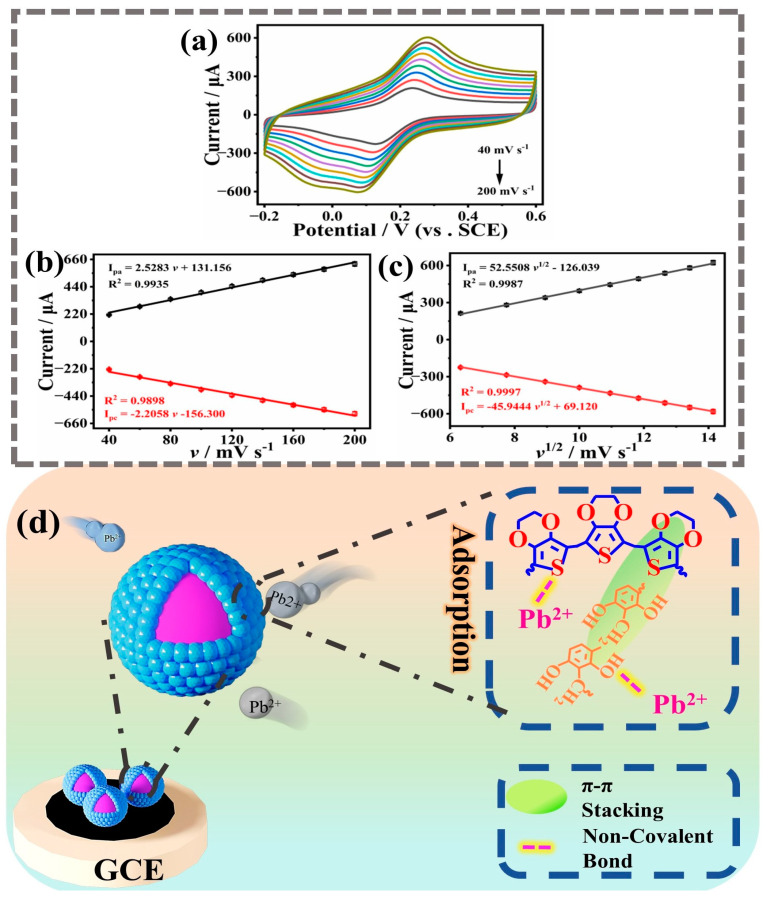
CV curves at different scan rates (**a**). Linear relationship between scan rate and peak current (**b**). Linear association between square root of scan rate and peak current (**c**). Pb^2+^ action mechanism (**d**).

The kinetics of electrode reactions are typically inferred from the relationships among the peak current, peak potential, and scan rate [40]. Figure 4a shows the CV curves for PEDOT/carbon sphere composites in a 5 mmol·L^−1^ K_3_[Fe(CN)_6_] solution that includes 0.1 mol·L^−1^ KCl. The curves were recorded at a range of scan rates, specifically between 40 mV s^−1^ and 200 mV s^−1^, highlighting the electrochemical behavior of the electrodes under different scanning conditions. This analysis yielded significant insights into the electrochemical properties of the materials and their reaction kinetics in various operational environments. Clearly, the peak REDOX current shows an increase with higher scanning rates. The peak currents, I_pa_ for the anode and I_pc_ for the cathode, exhibit a linear relationship with the scanning rate. It can be found in Figure 4b,c that the peak current of the PEDOT/carbon sphere is linear with the scanning rate (v) and the square root of the scanning rate (v^1/2^), respectively, indicating that there is a mixed control process of adsorption or diffusion on the electrode surface [41]. According to the linear relationship between its Ip and v^1/2^, I_pa_ = 52.5508 v^1/2^ − 126.039 (R^2^ = 0.9987), and I_pc_ = −45.9444 v^1/2^ + 69.120 (R^2^ = 0.9997); the electroactive surface area of PEDOT/carbon sphere/GCE is determined using the Randles–Sevcik equation [42].

(1) $$I_{p}={268,600n}^{3/2}{AD}^{1/2}{Cv}^{1/2}$$

In this context, $I_{p}$ represents the peak current, and n denotes the number of electrons that are transferred during the REDOX reaction in the potassium ferricyanide solution, with n being equal to 1. A refers to the electroactive surface area within the sensing system, D and C represent the diffusion coefficient and concentration of potassium ferricyanide, with values of 0.670 × 10^−5^ cm^2^ s^−1^ and 5.0 mM, and the scan rate is indicated by v. Therefore, the electroactive surface area of PEDOT/carbon sphere/GCE is 0.478 cm^2^, which suggests that the significant electroactive surface area of the sensor offers an effective platform for efficient Pb^2+^ adsorption and can support more recognition sites. As shown in Figure 4d, the interaction between PEDOT and the carbon sphere is enhanced through π-π stacking, leading to improved stability of the material. Simultaneously, the large surface area of the carbon sphere offers additional active sites, while the hydroxyl functional groups (-OH) on its surface can form non-covalent bonds with the sulfur atoms in the main chain of PEDOT. This interaction enhances the composite’s sensitivity and selectivity towards Pb^2+^ ions.

### 2.5. Sensing Performance Analysis

**Figure 5:**
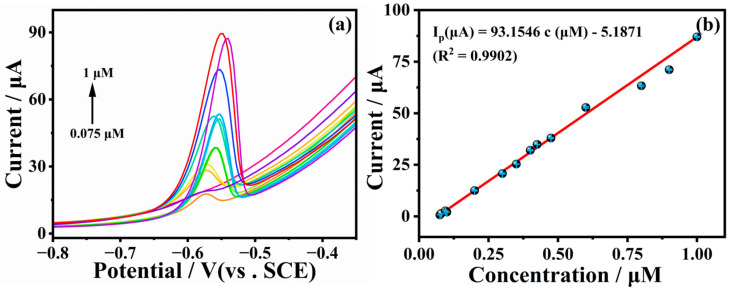
DPV response of PEDOT/carbon sphere/GCE with different concentrations of Pb^2+^ in ABS (pH = 5) (**a**) and calibration curve (**b**).

Under optimal experimental conditions (i.e., with the electrode being modified with PEDOT and carbon spheres in a 5:1 molar ratio, an ABS solution with a pH of 5, and a deposition time of 480 s), we conducted a DPV test for Pb^2+^. Figure 5a illustrates that the current response of Pb^2+^ increases linearly across a concentration range of 0.075 to 1.0 μM. Additionally, Figure 5b highlights a strong linear correlation between the current response to Pb^2+^ and its concentration, which is represented by the corresponding equation: I_p_ (μA) = 89.8327c − 5.4412 (R^2^ = 0.9918). Dependent on the 3σ value of the blank, we calculated the detection limit (LOD, S/N = 3) to be 0.035 nM, where σ represents the standard deviation of the sensor’s current response in blank ABS (n = 15). This suggests that the sensor shows remarkable sensitivity to Pb^2+^, featuring a broad linear range and a low detection limit.

By comparing the detection performance of Pb^2+^ with sensors reported by others, as shown in Table 1, the PEDOT/carbon sphere/GCE sensor that was studied in this work demonstrates greater sensitivity, a broader linear range, and reduced detection limits for Pb^2+^.

**Figure 6:**
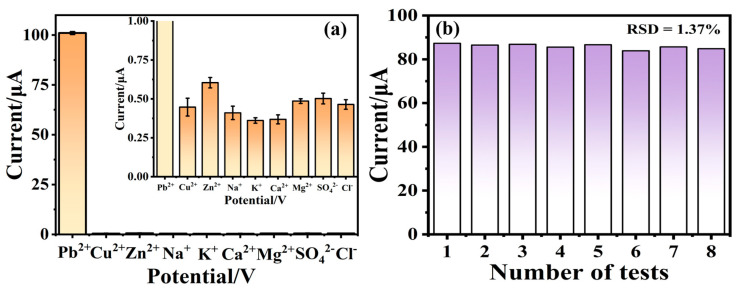
Current response of PEDOT/carbon sphere/GCE to Pb^2+^ (1 μM) and its potentially interfering ions (10 μM) (**a**); DPV response value of 1 μM Pb^2+^ for 9 consecutive assays on same PEDOT/carbon sphere/GCE (**b**).

The precise selectivity of the analyzed substance is a crucial criterion for the practical application value of the newly developed electrochemical sensor. In this selective experiment, PEDOT/carbon sphere/GCE was used to perform DPV tests on different ions (Cu^2+^, Zn^2+^, Na^+^, K^+^, Ca^2+^, Mg^2+^, SO_4_^2−^, Cl^−^), with ten times the concentration of Pb^2+^ being used as the experimental conditions. It is evident from Figure 6 that at the location of the current response signal for Pb^2^⁺, the current response signals of Cu^2+^, Zn^2+^, Na^+^, K^+^, Ca^2+^, Mg^2+^, SO_4_^2−^, and Cl^−^ are very low. This may be because the reduction potential of the selected enrichment and deposition of the tested metal ion (Pb^2+^) is −0.58~−0.56 V, and the selective oxidation of Pb^2+^ is further limited by the selected oxidation potential during the oxidation process of applying reverse voltage. Other ions do not interfere with this REDOX process. The experimental findings indicate that the PEDOT/carbon sphere/GCE exhibits good resistance to interference when detecting Pb^2+^.

For repeatability testing, the same sensor was analyzed ten times. It can be regenerated by rinsing with a mild acidic solution (0.1 M HAc for 10 min), which desorbs Pb^2^⁺ ions from the PEDOT surface without damaging the polymer structure. As shown in Figure 6a, the sensor RSD = 1.37% of its initial response after eight regeneration cycles, with only a slight decrease in sensitivity.

## 3. Experimental Procedure

### 3.1. Reagents

Poly(3,4-ethylenedioxythiophene) (EDOT) and resorcinol (C_6_H_6_O_2_, purity 99%) were supplied by Shanghai Aladdin Co., Ltd. (Shanghai, China). Formaldehyde (CH_2_O) and sodium acetate (C_2_H_3_O_2_Na) were acquired from Tianjin Zhiyuan Chemical Reagent Co., Ltd. (Tianjin, China). Acetic acid (C_2_H_4_O_2_) was purchased from Tianjin Xibote Chemical Co., Ltd. (Tianjin, China). Concentrated ammonia (H_5_NO) was sourced from Tianjin Baishi Chemical Co., Ltd. (Tianjin, China). Lead nitrate (Pb(NO_3_)_2_) and chloroform (CHCl_3_) were both acquired from Sichuan Xilong Scientific Co., Ltd. (Chengdu, China). And anhydrous ferric chloride (FeCl_3_) was purchased from Shanghai Macklin Inc. (Shanghai, China). Except for FeCl_3_, all solvents and reagents acquired were of an analytical quality. A background electrolyte consisting of an acetate buffer solution (ABS, 0.1 mol L^−1^) with a pH of 5.0 was used in the experiment.

### 3.2. Instruments

Measurements in electrochemistry were performed with a CHI 660C electrochemical workstation provided by Shanghai Chenhua Instrument Co., Ltd. (Shanghai, China). The electrochemical setup consists of a three-electrode configuration: The auxiliary electrode is made of platinum wire, while the reference electrode is a saturated calomel electrode (SCE). The working electrode can be either a bare carbon electrode or a modified glass carbon electrode (GCE). All components were obtained from Shanghai Yifeng Scientific Instrument Co., Ltd. (Shanghai, China). FTIR measurements were conducted with a Bruker Vertex 70 spectrometer. SEM images were obtained using a field-emission electron microscope (SU8220, Hitachi, Japan). XRD evaluation was conducted using a Bruker AXS X-ray diffractometer (D8 Advance) from Germany. Carbon materials were calcined using a tube furnace (GSL-1100X) provided by Hefei Kejing Materials Technology Co., Ltd. (Hefei, China).

### 3.3. Synthesis of Carbon Sphere

The specific preparation process of carbon balls was as follows: First, anhydrous ethanol (70.0 mL), distilled water (10.0 mL), and concentrated ammonia water (3.0 mL) were prepared into a mixed solution. After a 15 min blending period, phloroglucinol (0.4 g) and formaldehyde (0.56 mL) were added to the previously prepared solution. The mixture was then stirred continuously for 24 h. Upon completion of the reaction, the phenolic resin polymer precursor was obtained by washing it three times with anhydrous ethanol, followed by three washes with distilled water. Finally, the polymer precursor underwent vacuum dehydration at 60 °C for 12 h to remove any residual moisture. After the sample was completely dried, it was ground in a mortar and calcined at 700 °C under a nitrogen atmosphere for 5 h.

### 3.4. Synthesis of PEDOT/Carbon Sphere Composites

The PEDOT/carbon sphere composites were prepared by in situ oxidation polymerization. First, CHCl_3_ (40.0 mL) was slowly added to a polymer bottle containing solid carbon balls (0.013 g) and continuously agitated at room temperature for 30 min to make it evenly dispersed. PEDOT (0.057 g) was then dropped into the suspension, and anhydrous ferric chloride (0.03 g) was slowly added to the suspension, agitating it at room temperature for 48 h. The product was then rinsed in succession with trichloromethane, anhydrous ethanol, and distilled water until the filtrate became colorless. After undergoing freeze-drying for 24 h, a black solid powder was obtained, identified as PEDOT/carbon spheres. In addition, 1:1 PEDOT/carbon sphere, 2.5:1 PEDOT/carbon sphere, and 7.5:1 PEDOT/carbon sphere composites were prepared by the same method and steps.

### 3.5. Preparation of PEDOT/Carbon Sphere/GCE Sensor

**Scheme 1:**
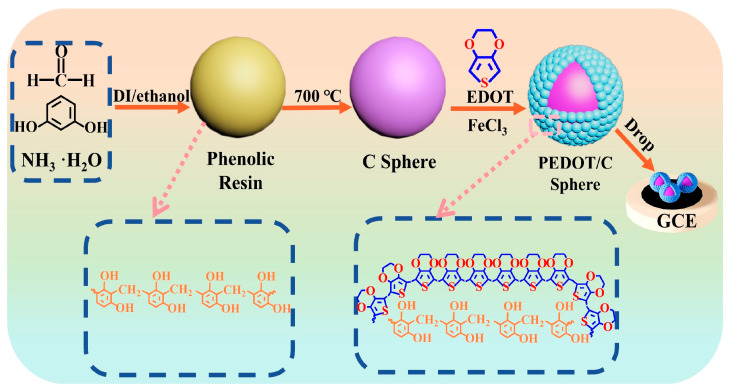
PEDOT/carbon sphere/GCE sensor preparation process.

First, polish the glassy carbon electrode (GCE) using α-alumina powder until the surface is smooth. Subsequently, wash the GCE sequentially with ultrapure water, a 50% (*v*/*v*) nitric acid solution, and a 50% (*v*/*v*) isopropanol solution to ensure thorough cleanliness. Next, uniformly apply 10.0 μL of a 1 mg/mL PEDOT/carbon sphere suspension onto the purified GCE and permit it to air-dry at room temperature. The resulting PEDOT/carbon sphere/GCE sensor is then ready for further use. The PEDOT/carbon sphere/GCE sensor preparation process is shown in Scheme 1.

### 3.6. Electrochemical Measurements

Electrochemical tests were conducted using an electrolyte solution containing 5 mM K_3_[Fe(CN)_6_]^3−/4−^ and 0.1 M KCl. The CV was performed within a potential range of −0.2 V to 0.6 V, with the scan rate set to 50 mV·s^−1^. For the EIS test, an amplitude of 5 mV was used, with a frequency range from 0.01 Hz to 100 kHz. Differential pulse voltammetry (DPV) was performed using an acetoacetate sodium acetate (ABS) buffer. The potential range for the test was set between −0.2 V and −1 V, with a scan rate of 50 mV·s^−1^. The pulse parameters included an amplitude of 0.05 V, a duration of 0.05 s, and an interval of 0.1 s.

## 4. Conclusions

In this study, a PEDOT/carbon sphere composite was synthesized through in situ oxidative polymerization and utilized for the electrochemical detection of Pb^2^⁺, and a PEDOT/carbon sphere/GCE electrochemical sensor was constructed. Because of the composite with a carbon sphere, PEDOT can be uniformly dispersed on the carrier, which can reduce its agglomeration, thus significantly enhancing its chemical stability and electrochemical activity. Secondly, the sulfur atoms in the PEDOT main chain and the hydroxyl functional groups in the solid carbon balls can interact with Pb^2+^ to significantly enhance the sensitivity and selectivity of the modified electrode, facilitating a high adsorption capacity when analyzing Pb^2+^ through electrochemical methods. The architecture and morphology of the PEDOT/carbon sphere composite materials were analyzed, and their electrochemical properties were tested to analyze their electrochemical behavior. Under optimal conditions, the sensor demonstrated an impressive linear response range of 0.075 to 1.0 μM and a low detection limit of 0.035 nM. It also exhibited high selectivity for detecting Pb^2^⁺, effectively functioning in the presence of interfering ions such as Cu^2+^, Zn^2+^, Na^+^, K^+^, Ca^2+^, Mg^2+^, SO_4_^2−^, and Cl^−^. This capability makes it suitable for applications in environmental monitoring, food safety, and biomedical fields for Pb^2+^ detection. In PEDOT/CS composites, a hydroxyl group (-OH) on the surface of carbon spheres can improve the hydrophilicity of the composites. However, this kind of hydrophilic improvement still has some limitations. It is hoped that in the future, materials with better hydrophilicity can be developed and applied to the detection of heavy metal ions.

## Figures and Tables

**Table 1:** Comparison of Pb^2+^ performances measured by various sensors.

Sensor	Detection Method	Linearity Range	Detection Limit	Reference
BFS	DPV	1~5 μM	1.5 × 10^−2^ μM	[43]
PA−PANI	DPSAV	0.01~6 mM	5.0 μM	[44]
P1,2-DAAQ	SWASV	0~12 mM	58 μM	[45]
D−BN	LSASV	0.05~40 mM	15 μM	[46]
CuONPs/PANI–CPE	SWV	0.01~0.14 μM	4.8 × 10^−3^ μM	[47]
PEDOT/C Sphere	DPV	0.075~1 μM	3.5 × 10^−5^ μM	This work

## Data Availability

Data will be made available on request.

## References

[1] Malik L.A., Bashir A., Qureashi A., Pandith A.H. (2019). Detection and removal of heavy metal ions: A review. Environ. Chem. Lett..

[2] Bolisetty S., Peydayesh M., Mezzenga R. (2019). Sustainable technologies for water purification from heavy metals: Review and analysis. Chem. Soc. Rev..

[3] Nguyen H., Sung Y., O’Shaughnessy K., Shan X., Shih W.-C. (2018). Smartphone Nanocolorimetry for On-Demand Lead Detection and Quantitation in Drinking Water. Anal. Chem..

[4] Jiang H., Ji P., Xu Y., Liu X., Kong D. (2021). Self-paired dumbbell DNA-assisted simple preparation of stable circular DNAzyme and its application in Pb^2+^ sensor. Anal. Chim. Acta.

[5] Gao S., Xu C., Yalikun N., Mamat X., Li Y., Wågberg T., Hu X., Liu J., Luo J., Hu G. (2017). Sensitive and Selective Differential Pulse Voltammetry Detection of Cd(II) and Pb(II) Using Nitrogen-Doped Porous Carbon Nanofiber Film Electrode. J. Electrochem. Soc..

[6] Wang G., Sun D., Liang L., Wang G., Ma J. (2023). Highly sensitive detection of trace lead ions concentration based on a functional film-enhanced optical microfiber sensor. Opt. Laser Technol..

[7] Boruah B.S., Biswas R. (2021). In-situ sensing of hazardous heavy metal ions through an ecofriendly scheme. Opt. Laser Technol..

[8] Haixia S., Zaijun L., Ming L. (2007). Ionic liquid 1-octyl-3-methylimidazolium hexafluorophosphate as a solvent for extraction of lead in environmental water samples with detection by graphite furnace atomic absorption spectrometry. Microchim. Acta.

[9] Ajab H., Ali Khan A.A., Nazir M.S., Yaqub A., Abdullah M.A. (2019). Cellulose-hydroxyapatite carbon electrode composite for trace plumbum ions detection in aqueous and palm oil mill effluent: Interference, optimization and validation studies. Environ. Res..

[10] Duan S., Wu X., Gong Z., Wang J., Liu X., Wang Q., Wang Y., Dai H. (2024). Curcumin-based ratiometric electrochemical sensing interface for the detection of Cd^2+^ and Pb^2+^ in grain products. Colloids Surf. A.

[11] Li J., Lu F., Umemura T., Tsunoda K.-I. (2000). Determination of lead by hydride generation inductively coupled plasma mass spectrometry. Anal. Chim. Acta.

[12] Londonio A., Morzán E., Smichowski P. (2019). Determination of toxic and potentially toxic elements in rice and rice-based products by inductively coupled plasma-mass spectrometry. Food Chem..

[13] Noudeng V., Pheakdey D.V., Xuan T.D. (2024). Toxic heavy metals in a landfill environment (Vientiane, Laos): Fish species and associated health risk assessment. Environ. Toxicol. Pharmacol..

[14] Pu H., Ruan S., Yin M., Sun Q., Zhang Y., Gao P., Liang X., Yin W., Fa H.-B. (2022). Performance comparison of simultaneo us detection Heavy-Metal ions based on carbon materials electrochemical sensor. Microchem. J..

[15] Song H., Huo M., Zhou M., Chang H., Li J., Zhang Q., Fang Y., Wang H., Zhang D. (2024). Carbon nanomaterials-based electrochemical sensors for heavy metal detection. Crit. Rev. Anal. Chem..

[16] Li B., Xie X., Meng T., Guo X., Li Q., Yang Y., Jin H., Jin C., Meng X., Pang H. (2024). Recent advance of nanomaterials modified electrochemical sensors in the detection of heavy metal ions in food and water. Food Chem..

[17] Meng R., Zhu Q., Long T., He X., Luo Z., Gu R., Wang W., Xiang P. (2023). The innovative and accurate detection of heavy metals in foods: A critical review on electrochemical sensors. Food Control..

[18] Yang M., Li P.H., Chen S.H., Xiao X.Y., Tang X.H., Lin C.H., Huang X.J., Liu W.Q. (2020). Nanometal oxides with special surface physicochemical properties to promote electrochemical detection of heavy metal ions. Small.

[19] Lee S., Oh J., Kim D., Piao Y. (2016). A sensitive electrochemical sensor using an iron oxide/graphene composite for the simultaneous detection of heavy metal ions. Talanta.

[20] Tang K., Chen Y., Zhao Y. (2024). Exploiting halide perovskites for heavy metal ion detection. Chem. Commun..

[21] Wang Y., Wang L., Huang W., Zhang T., Hu X., Perman J.A., Ma S. (2017). A metal–organic framework and conducting polymer based electrochemical sensor for high performance cadmium ion detection. J. Mater. Chem. A.

[22] Meenakshi S., Kaladevi G., Pandian K., Gopinath S.C. (2024). Metal-polymer-clay nanocomposites based electrochemical sensor for detecting hydrazine in water sources. Colloids Surf. A.

[23] Dietrich M., Heinze J., Heywang G., Jonas F. (1994). Electrochemical and spectroscopic characterization of polyalkylenedioxythiophenes. J. Electroanal. Chem..

[24] Groenendaal L., Jonas F., Freitag D., Pielartzik H., Reynolds J.R. (2000). Poly(3,4-ethylenedioxythiophene) and its derivatives: Past, present, and future. Adv. Mater..

[25] Qian Y., Ma C., Zhang S., Gao J., Liu M., Xie K., Wang S., Sun K., Song H. (2018). High performance electrochemical electrode based on polymeric composite film for sensing of dopamine and catechol. Sensor Actuat. B Chem..

[26] Leprince M., Regal S., Mailley P., Sauter-Starace F., Texier I., Auzély-Velty R. (2023). A cross-linkable and resorbable PEDOT-based ink using a hyaluronic acid derivative as dopant for flexible bioelectronic devices. Mater. Adv..

[27] Huang C.-H., Chen Z.-Y., Chiu C.-L., Huang T.-T., Meng H.-F., Yu P. (2019). Surface micro-/nanotextured hybrid PEDOT: PSS-silicon photovoltaic cells employing Kirigami graphene. ACS Appl. Mater. Interfaces.

[28] Tian Q., Xu J., Zuo Y., Li Y., Zhang J., Zhou Y., Duan X., Lu L., Jia H., Xu Q. (2019). Three-dimensional PEDOT composite based electrochemical sensor for sensitive detection of chlorophenol. J. Electroanal. Chem..

[29] Lu X., Li Y., Duan X., Zhu Y., Xue T., Rao L., Wen Y., Tian Q., Cai Y., Xu Q. (2021). A novel nanozyme comprised of electro-synthesized molecularly imprinted conducting PEDOT nanocomposite with graphene-like MoS_2_ for electrochemical sensing of luteolin. Microchem. J..

[30] Meng R., Tang J., Wu X., Zhang S., Wang X., Li Q., Jin R. (2021). Molecularly imprinted electrochemical sensor based on 3D wormlike PEDOT-PPy polymer for the sensitive determination of rutin in flos sophorae immaturus. J. Electrochem. Soc..

[31] Zhou H., Liu G., Liu J., Wang Y., Ai Q., Huang J., Yuan Z., Tan L., Chen Y. (2017). Effective network formation of pedot by in-situ polymerization using novel organic template and nanocomposite supercapacitor. Electrochim. Acta.

[32] Yao Y., Xu J., Huang Y., Zhang T. (2024). Synthesis and applications of carbon nanospheres: A review. Particuology.

[33] Zhang L., Liu L., Liu M., Yu Y., Hu Z., Liu B., Lv H., Chen A. (2019). Controllable synthesis of N-doped hollow, yolk-shell and solid carbon spheres via template-free method. J. Alloys Comp..

[34] Wang X., Zhou J., Xing W., Liu B., Zhang J., Lin H., Cui H., Zhuo S. (2017). Resorcinol–formaldehyde resin-based porous carbon spheres with high CO_2_ capture capacities. J. Energy Chem..

[35] Luan K., Yao S., Zhang Y., Zhuang R., Xiang J., Shen X., Li T., Xiao K., Qin S. (2017). Poly (3, 4-ethyleendioxythiophene) coated titanium dioxide nanoparticles in situ synthesis and their application for rechargeable lithium sulfur batteries. Electrochim. Acta.

[36] Chen S., Chen W., Wang Y., Wang X., Ding Y., Zhao D., Liu J. (2022). Facile one-pot method of AuNPs/PEDOT/CNT composites for simultaneous detection of dopamine with a high concentration of ascorbic acid and uric acid. RSC Advan..

[37] Ravit R., Abdullah J., Ahmad I., Sulaiman Y. (2019). Electrochemical performance of poly (3, 4-ethylenedioxythipohene)/nanocrystalline cellulose (PEDOT/NCC) film for supercapacitor. Carbohydr. Polym..

[38] Shin H.-J., Jeon S.S., Im S.S. (2011). CNT/PEDOT core/shell nanostructures as a counter electrode for dye-sensitized solar cells. Synth. Met..

[39] Zhou Y., Abdurexit A., Jamal R., Abdiryim T., Liu X., Liu F., Xu F., Zhang Y., Wang Z. (2024). Highly sensitive electrochemical sensing of norfloxacin by molecularly imprinted composite hollow spheres. Biosens. Bioelectron..

[40] Yang Y., Fang G., Liu G., Pan M., Wang X., Kong L., He X., Wang S. (2013). Electrochemical sensor based on molecularly imprinted polymer film via sol–gel technology and multi-walled carbon nanotubes-chitosan functional layer for sensitive determination of quinoxaline-2-carboxylic acid. Biosens. Bioelectron..

[41] Li G., Qi X., Wu J., Xu L., Wan X., Liu Y., Chen Y., Li Q. (2022). Ultrasensitive, label-free voltammetric determination of norfloxacin based on molecularly imprinted polymers and Au nanoparticle-functionalized black phosphorus nanosheet nanocomposite. J. Hazard. Mater..

[42] Wang Y., Yao L., Liu X., Cheng J., Liu W., Liu T., Sun M., Zhao L., Ding F., Lu Z. (2019). CuCo_2_O_4_/N-Doped CNTs loaded with molecularly imprinted polymer for electrochemical sensor: Preparation, characterization and detection of metronidazole. Biosens. Bioelectron..

[43] Mourya A., Mazumdar B., Sinha S.K. (2019). Determination and quantification of heavy metal ion by electrochemical method. J. Environ. Chem. Eng..

[44] Huang H., Zhu W., Gao X., Liu X., Ma H. (2016). Synthesis of a novel electrode material containing phytic acid-polyaniline nanofibers for simultaneous determination of cadmium and lead ions. Anal. Chim. Acta.

[45] Hassan K.M., Gaber S.E., Altahan M.F., Azzem M.A. (2018). Novel Sensor Based on Poly (1, 2-Diaminoanthraquinone) for Individual and Simultaneous Anodic Stripping Voltammetry of Cd^2+^, Pb^2+^, Cu^2+^ and Hg^2+^. Electroanalysis.

[46] Shen Y., Ouyang H., Li W., Long Y. (2021). Defect-enhanced electrochemical property of *h*-BN for Pb^2+^ detection. Microchim. Acta.

[47] Ali D.S.B., Krid F., Nacef M., Boussaha E.H., Chelaghmia M.L., Tabet H., Selaimia R., Atamnia A., Affoune A.M. (2023). Green synthesis of copper oxide nanoparticles using *Ficus elastica* extract for the electrochemical simultaneous detection of Cd^2+^, Pb^2+^, and Hg^2+^. RSC Adv..

